# European Network of Pregnancy Registers in Rheumatology (EuNeP)—an overview of procedures and data collection

**DOI:** 10.1186/s13075-019-2019-3

**Published:** 2019-11-14

**Authors:** Yvette Meissner, Anja Strangfeld, Nathalie Costedoat-Chalumeau, Frauke Förger, Doreen Goll, Anna Molto, Rebecca Özdemir, Marianne Wallenius, Rebecca Fischer-Betz

**Affiliations:** 1 Programmbereich Epidemiologie, Forschungsbereich Epidemiologie, Deutsches Rheuma-Forschungszentrum, Charitéplatz 1, 10117 Berlin, Germany; 2 Assistance Publique-Hôpitaux de Paris (AP-HP), Internal Medicine Department, Cochin Hospital, Referral Center for Rare Autoimmune and Systemic Diseases, Paris, France; 3 Paris Descartes Sorbonne, Paris Cité University, Paris, France; 4 INSERM (Unit 1153) Université de Paris, CRESS, Paris, France; 5 Department of Rheumatology, Inselspital University Hospital, Bern, Switzerland; 6 Patient Partner, Berlin, Germany; 7 Assistance Publique-Hôpitaux de Paris (AP-HP), Rheumatology Department, Cochin Hospital, Paris, France; 8 Patient Partner, Duisburg, Germany; 9 National Advisory Unit on Pregnancy and Rheumatic Diseases, Department of Rheumatology, Saint Olavs University Hospital in Trondheim, Trondheim, Norway; 10 Department of Neuromedicine and Movement Science, Norwegian University of Science and Technology, Trondheim, Norway; 11 Department of Rheumatology and Hiller Research Unit, University Clinic Duesseldorf, Duesseldorf, Germany

**Keywords:** Inflammatory rheumatic diseases, Pregnancy, Cohort study, Registry, Outcome measures, Outcomes research, Data harmonization

## Abstract

**Background:**

The collaborative initiative of the European Network of Pregnancy Registers in Rheumatology (EuNeP) aims to combine data available in nationwide pregnancy registers to increase knowledge on pregnancy outcomes in women with inflammatory rheumatic diseases (IRD) and on drug safety during pregnancy and lactation. The objective of this study was to describe the similarities and differences of the member registers.

**Methods:**

From all registers, information about their structure and design was collected, as well as which parameters regarding demographics, maternal outcomes, treatment, course and outcome of pregnancy, and development of the child were available in the respective datasets. Furthermore, the current recruitment status was reported.

**Results:**

The four registers (EGR2 (France), RePreg (Switzerland), RevNatus (Norway), and Rhekiss (Germany)) collect information prospectively and nationwide. Patients can be enrolled before conception or during pregnancy. To date, more than 3500 patients in total have been included, and data on 2200 pregnancies with an outcome are available. The distribution of diagnoses in the respective registers varies considerably, and only three entities (rheumatoid arthritis, psoriatic arthritis, and spondyloarthritis) are captured by all the registers. Broad consistency was found in non-disease-specific data items, but differences regarding instruments and categories as well as frequency of data collection were revealed. Disease-specific data items are less homogeneously collected.

**Conclusion:**

Although the registers in this collaboration have similar designs, we found numerous differences in the variables collected. This survey of the status quo of current pregnancy registers is the first step towards identifying data collected uniformly across registers in order to facilitate joint analyses.

**Trial registration:**

Not applicable.

## Background

Inflammatory rheumatic diseases (IRDs) frequently affect women of childbearing age and often interfere with family planning, a period that is characterized by concerns and conflicting information for both patients and physicians. Within the last decades and the increasing treatment possibilities to maintain inflammatory activity under control, the awareness of reproduction in rheumatic diseases has been growing [1]. Although recommendations have been established for the management and treatment of IRD patients with a wish for a child or during pregnancy [2, 3], there is still limited evidence-based information on the influence of IRD and its drug treatment on the course and outcome of pregnancy.

In 2018, the National Institutes of Health (NIH)-led Task Force on Research Specific to Pregnant Women and Lactating Women (PRGLAC) recommended optimizing research in pregnant women and developing disease- or condition-focused registers and standards [4]. Prospective pregnancy registers offer systematic data collection on pre-specified outcomes and can provide a wealth of information. With regular follow-up during pregnancy and detailed information on disease activity and treatment status at several time points, the results obtained go far beyond what can be collected from administrative databases or global safety registers. However, collecting data on a large enough set of pregnancies in each of these individual registers is a challenge. For example, the MotherToBaby study on the safety of exposure to adalimumab during early pregnancy needed approximately 10 years to reach a sample size of 74 women with exposure to the tumour necrosis factor (TNF) inhibitor during pregnancy [5]. Similarly, accrual within the ‘Predictors of Pregnancy Outcome: Biomarkers in Antiphospholipid Antibody Syndrome and Systemic Lupus Erythematosus’ (PROMISSE) study remained very slow, taking a decade to enrol almost 350 lupus pregnancies [6].

Recently, the European League Against Rheumatism (EULAR) strengthened the need for future research in the area of IRD and reproductive health issues, recommending joint approaches among several countries to enable collaborative data analyses [3]. One step towards collaborative analyses was completed by initiating the European Network of Pregnancy Registers in Rheumatology (EuNeP) that started in September 2017.

As a first task within the EuNeP collaboration, we aimed to evaluate the status quo of the participating registers, as we think this information is a prerequisite for collaborative research. Thus, the objective of this work was to examine the study designs and structures of the involved registers, considering the items and extent of data collection. In addition, an overview of selected characteristics of patients and pregnancies currently included in all registers is provided.

## Methods

### Data examination

A comprehensive survey was performed on details about all registers participating in the EuNeP collaboration. The survey was carried out using a three-step process. First, a questionnaire was developed to request general information about the registers and to examine the following categories of data collection: patient enrolment and inclusion criteria, demographics, disease-specific outcomes including immunology, laboratory markers and disease activity, non-disease-specific outcomes such as adverse events and hospital admissions, medical treatment, course and outcome of pregnancy, and outcomes of the child. If certain data items were collected, it was asked for the person reporting the information (physician/patient), for the time points and frequency of data collection during child wish, pregnancy, and postpartum. In addition, instruments or categories used to collect the information were reported. All questions were equipped with free text space for additional information or in case the correct answer was not included as an option.

All additional data items reported via free text were circulated in a second round, and all other registers were asked whether they also collect the corresponding item. Finally, each register provided information on its current recruitment status, the distribution of diagnoses, the baseline characteristics of patients enrolled so far, and the number of pregnancies observed. All answers were filled in a template by the principle investigator(s) of the member registers. The completed tables were reviewed and queried in case of uncertainties.

### Statistical analyses

All responses were evaluated descriptively.

## Results

The registers involved in the EuNeP project are EGR2 (France), RePreg (Switzerland), RevNatus (Norway), and Rhekiss (Germany). All three phases of the survey were completed by representatives of the four registers, and outstanding questions were answered.

### Study design of registers collaborating in EuNeP

All registers are established nationwide and were founded in recent years, except for RevNatus, which started in 2006 (Table 1). While EGR2 and Rhekiss were founded as independent registers, the Swiss RePreg is attached to the Swiss Clinical Quality Management in Rheumatic Diseases (SCQM) register, and information concerning the underlying diseases is drawn from study visits reported in SCQM.

**Table 1:** General information and study design of registers collaborating in the EuNeP project

	EGR2	RePreg	RevNatus	Rhekiss
General information				
Country	France (FR)	Switzerland (CH)	Norway (NO)	Germany (DE)
Founding year	2014	2017	2006	2015
Data reported by	Physician (rheumatologist, internist, others^1^), study nurse^2^, patient^2,3^	Rheumatologist, study nurse, patient	Rheumatologist, study nurse, patient	Rheumatologist, study nurse, patient
Way of data reporting	Web-based (paper-based^2^)	Web-based	Web-based (paper-based until 2016)	Web-based, app for smartphones and tablets (only for patients)
Time of enrolment and frequency of visits				
Child wish/pre-conception				
Enrolment possible	Yes	Yes	Yes	Yes
Maximum number of visits	8	1	1	5
Maximum observation time	96 months	6 months	12 months	24 months
Frequency of data collection	Every 12 months	–	–	Every 6 months
Pregnancy				
Enrolment possible (up to WG)	Yes (12^4^)	Yes (complete pregnancy)	Yes (complete pregnancy)	Yes (20)
Maximum number of visits	Unlimited	3	3	3
Maximum observation time	Complete pregnancy	Complete pregnancy	Complete pregnancy	Complete pregnancy
Frequency of data collection	At least once every trimester	Once every trimester	Once every trimester	Once every trimester
Postpartum				
Enrolment possible	No	Yes^5^	No	No
Maximum number of visits	2	5	3	4
Maximum observation time	12 months	48 months	12 months	24 months
Frequency of data collection	Month 6, 12 pp	Month 2, 6, 12, 24, 48 pp	Week 6, month 6, 12 pp	Month 6, 12, 18, 24 pp

*Abbreviations*: *pp* postpartum, *WG* week of gestation

^1^Internal medical specialists, obstetricians, and physicians of other specialities

^2^Data is always validated by a physician

^3^Patients fill out paper questionnaires, which are then entered into eCRFs by study coordinator

^4^Enrolment after WG 12 is possible in some specific cases

^5^Enrolment is possible until week 8 postpartum

All registers cover data of women with IRD before, during, and after pregnancy as well as data on child development. Notably, none of the registers collects data for men with IRD and a wish for a child, for men becoming a father, or of a healthy control group. Data are reported prospectively by physicians or study nurses and by patients of multiple centres. In all registers, patients can be included before conception (child wish) or during pregnancy. In addition, the Swiss register RePreg accepts registration of patients up to week 8 after birth. Women can participate with multiple pregnancies and with subsequent pregnancies in the four registers.

The study protocols of all registers were approved by the respective national ethics committees. Prior to enrolment, patients participating in the German, Norwegian, and Swiss register have to give written informed consent. In France, they precisely state their ‘non-opposition’ to the data collection. The individual registers have different funding strategies and receive financial support from various sources including grants from their home institute, national health organizations, and patient associations as well as local and industrial grants.

**Fig. 1:**
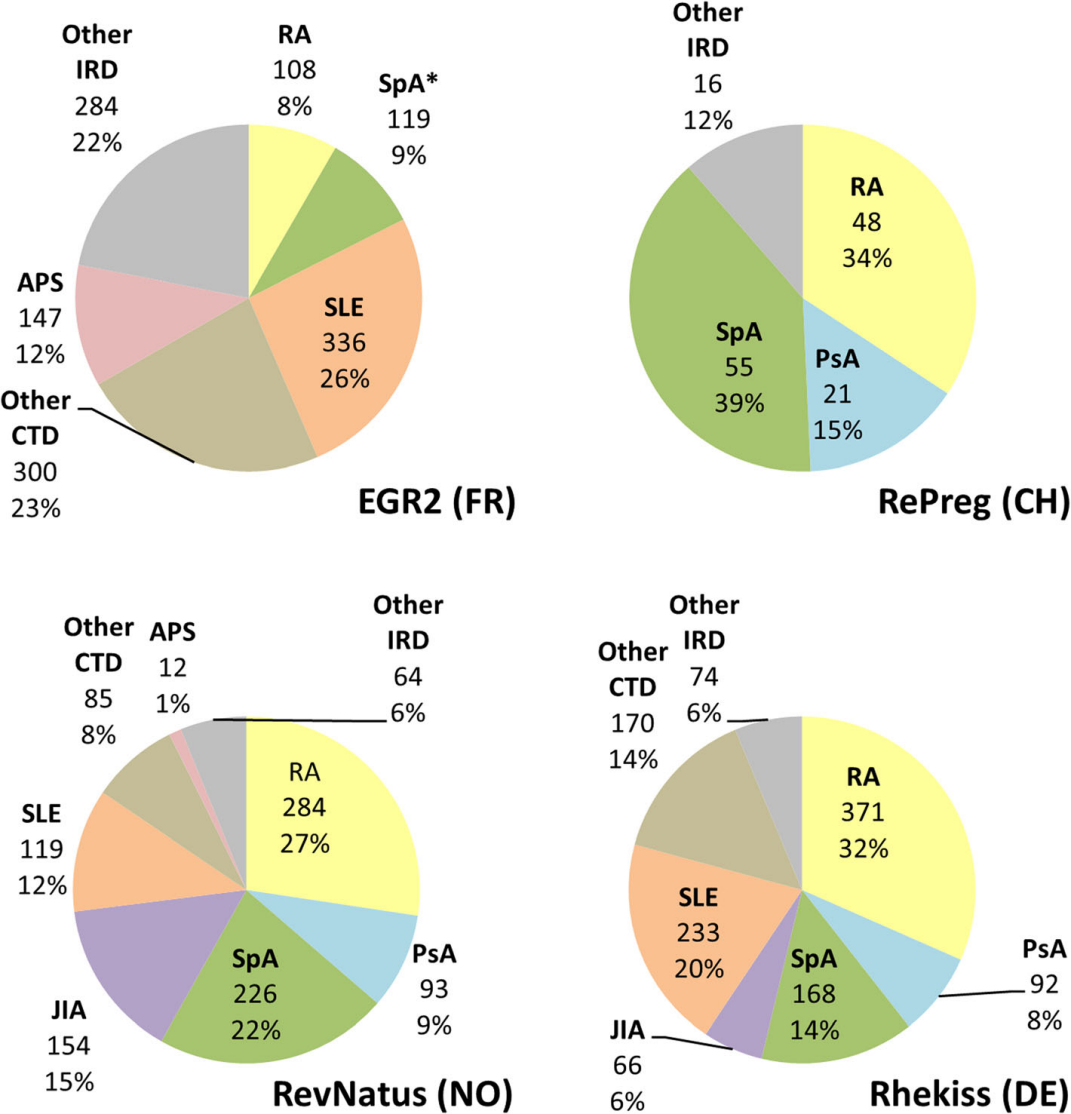
Distribution of diagnosis of enrolled patients in the registers collaborating in EuNeP. Figures represent diagnoses, number of included patients, and respective percentages. Patients can be enrolled several times since they can contribute more than one pregnancy. *PsA patients are included as peripheral SpA. RA, rheumatoid arthritis; PsA, psoriatic arthritis; SpA, spondyloarthritis; JIA, juvenile idiopathic arthritis; SLE, systemic lupus erythematosus; Other CTD, other connective tissue diseases; APS, primary antiphospholipid syndrome; Other IRD, other inflammatory rheumatic diseases (e.g. autoinflammatory diseases, Behcet’s disease, vasculitis, and other rare diseases)

All registers enrol women with a physician-confirmed diagnosis of various IRDs. Entities captured in all four registers encompass rheumatoid arthritis (RA), psoriatic arthritis (PsA), and spondyloarthritis (SpA). Three registers include patients with systemic lupus erythematosus (SLE), other connective tissue diseases (including Sjögren’s syndrome, scleroderma, myositis, and mixed connective tissue diseases), and vasculitis. Two registers also enrol patients with primary antiphospholipid syndrome, juvenile idiopathic arthritis (JIA), autoinflammatory diseases, Behcet’s disease, and other rare diseases such as mastocytosis. The distribution of IRD diagnoses of enrolled patients therefore differs among registers (Fig. 1). The number of patients enrolled in the individual registers, the observed and completed pregnancies, and the participating units are given in Table 2.

**Table 2:** Key numbers of registers participating in the EuNeP project

	EGR2 (FR)	RePreg (CH)	RevNatus* (NO)	Rhekiss (DE)
Database closed on	17 February 2019	01 February 2019	18 February 2019	03 January 2019
Enrolments (total)	1304	140	1037	1174
Thereof in child wish module	204	61	500	356
Thereof in pregnancy module	1091	71	537	818
Thereof in postpartal module	0	8	0	0
Thereof unknown	9	0	0	0
Observed pregnancies	1177	83	704	976
Thereof completed	843	67	645	689
Participating units	70	51	17	97

*Only patients enrolled since March 2016 in the IT-based system are considered for the analyses

### Items, details, and frequency of data collection

In general, data collection can be distinguished into data collected for all patients irrespective of the underlying IRD and data collected only for specific diseases.

**Fig. 2:**
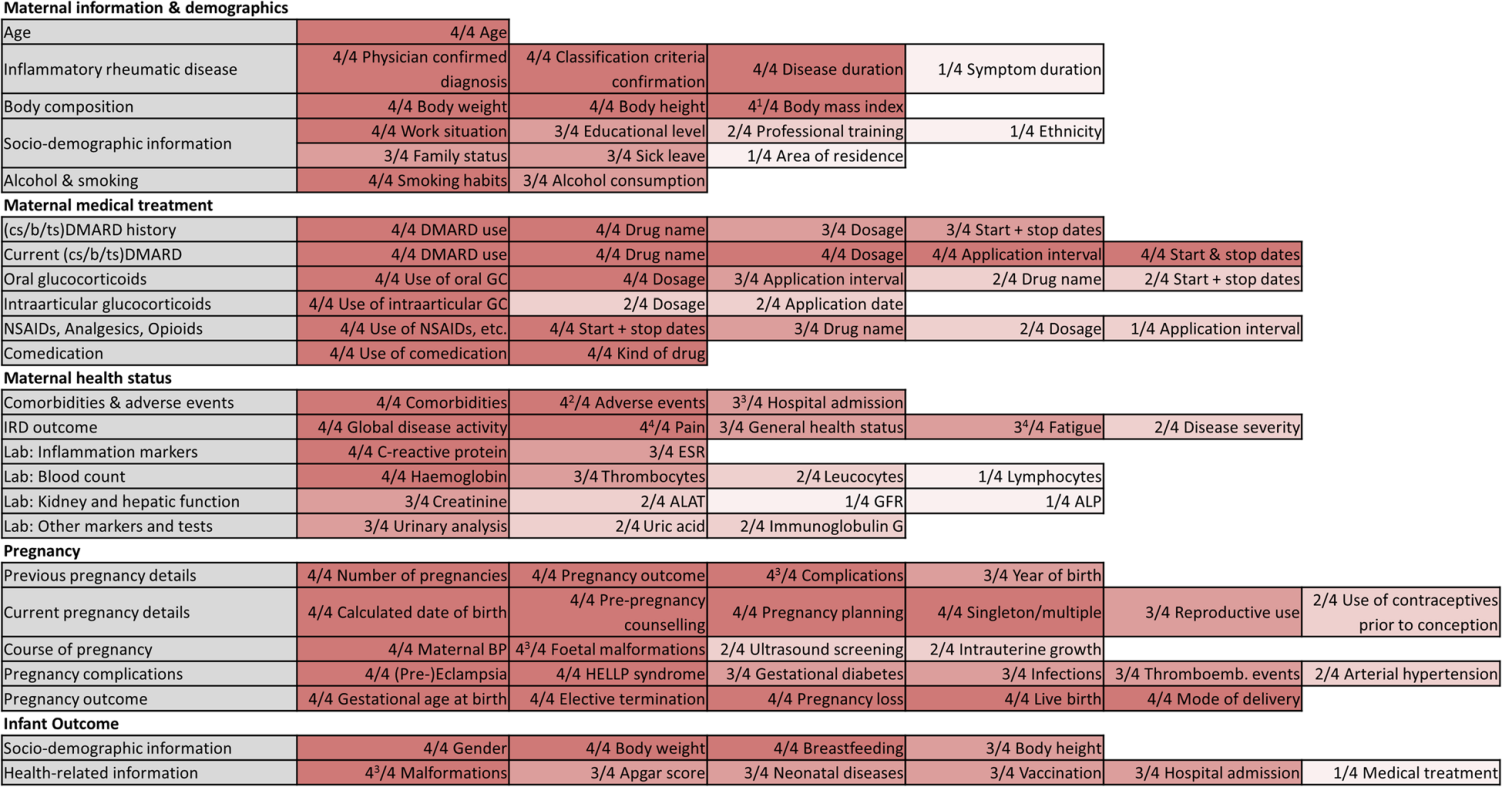
Data items reported for all patients in registers cooperating in EuNeP. Figure represents the number of registers that report the specific data item. ALAT, alanine aminotransferase; ALP, alkaline phosphatase; BP, blood pressure; (cs/b/ts)DMARD, (conventional synthetic/biologic/targeted synthetic) disease-modifying anti-rheumatic drug; ESR, erythrocyte sedimentation rate; GC, glucocorticoid; GFR, glomerular filtration rate; HELLP syndrome, Hemolysis, Elevated Liver, Low Platelets syndrome; IRD, inflammatory rheumatic disease; Lab, laboratory measures; NSAID, non-steroidal anti-inflammatory drug. ^1^In two registers, BMI is not directly reported but can be calculated. ^2^Adverse events are registered as change in comorbidity in one register. ^3^One register does not capture the data but can get information through linkage to other registers. ^4^Only for selected diseases in one register

A range of non-disease-specific data items are collected by all registers (Fig. 2). Some specifications are collected homogeneously across registers, for instance, treatment with disease modifying anti-rheumatic drugs (DMARDs) comprising the type of DMARD, dosage, application interval, and start and stop dates. For others, the consistency between the four registers decreases with respect to the details of data items: the use of non-steroidal anti-inflammatory drugs (NSAIDs) and their application dates are captured by all registers, but information on the active compound, dosage, and application intervals is only available in 3, 2, and 1 register(s), respectively. The Norwegian register does not capture some of the medical information directly, e.g. maternal hospital admission or neonatal malformations, but data can be retrieved by linkage to other registers such as the national birth register or the patient register via a unique patient identifier.

Evident differences were seen in the instruments used for data reporting (Additional file 1: Table S1). For instance, for patient-reported outcomes, either numeric rating scales, visual analogue scales, or—for the general health status—a question of the Short Form (SF) 12 patient questionnaire are used. Moreover, the characteristics of categorical variables varied. For example, in some registers, smoking is reported as smoking/not smoking during the past 3 months, while in others, it is reported as current/past/never and sometimes with details such as frequency of smoking, pack years, or date of stopping. The mode of delivery is categorized into spontaneous birth and Caesarean section (including elective and emergency Caesarean section) in all registers, and operative vaginal delivery is reported in two registers. For some variables such as educational level, categories differ according to national circumstances. Another variation was revealed in the reporting of comorbidities. While all registers collect comorbidities via pre-defined lists, one register has additional free text space. Although all four registers report adverse events (AEs), in one of the registers, the occurrence of AEs is only recorded via the change in comorbidities.

Differences were also found regarding the frequency of data collection. Items are either collected at patient enrolment, at regular visits, only during pregnancy, only after giving birth, or at pre-specified selected visits.

**Fig. 3:**
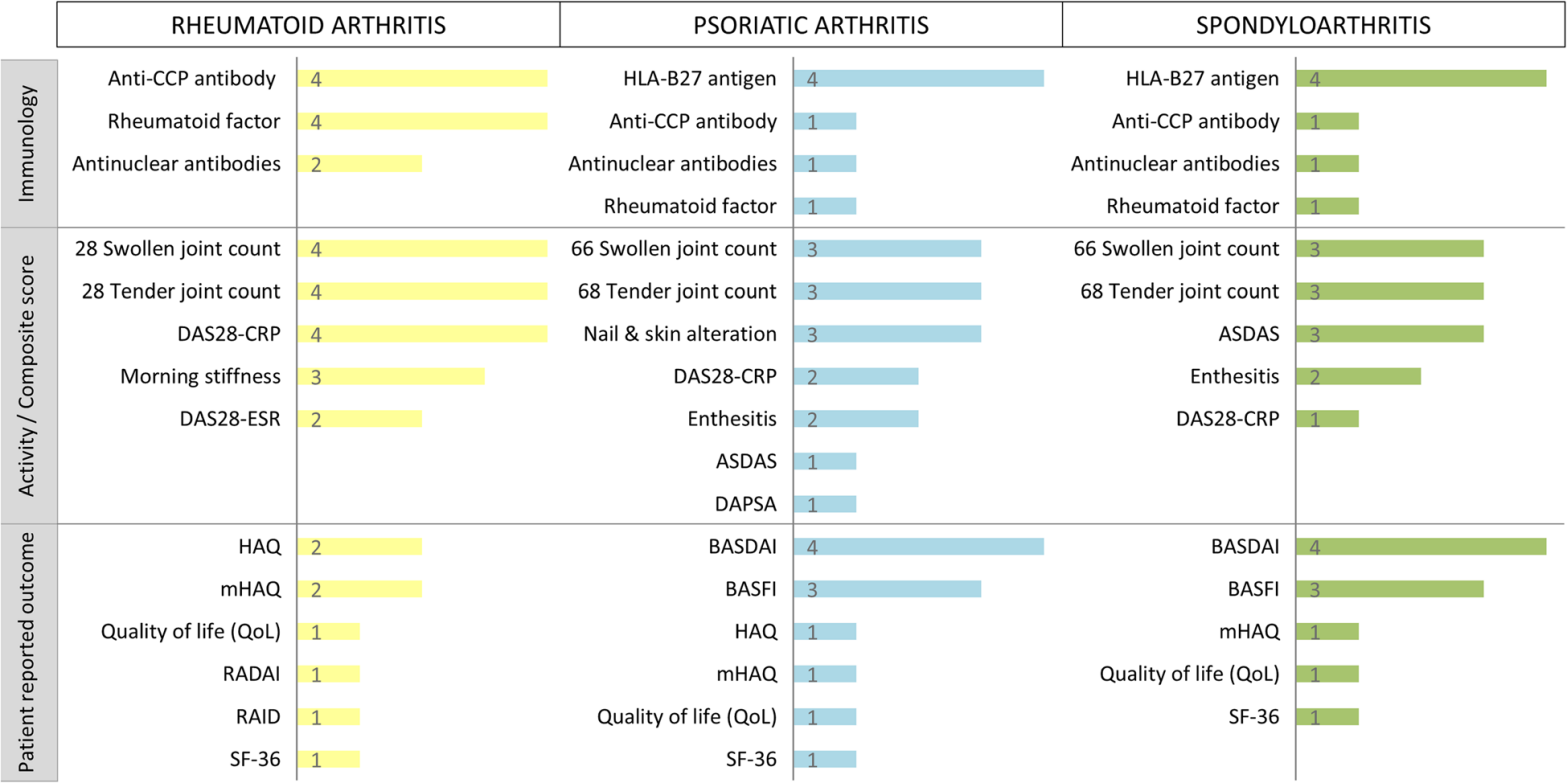
Frequency of data items collected for patients with rheumatoid arthritis, psoriatic arthritis, and spondyloarthritis. Figures in the bars indicate the numbers of registers in which this variable is collected. Anti-CCP antibody, anti-citrullinated protein antibody; ASDAS, Ankylosing Spondylitis Disease Activity Score; BASDAI, Bath Ankylosing Spondylitis Disease Activity Index; BASFI, Bath Ankylosing Spondylitis Functional Index; CRP, C-reactive protein; DAPSA, Disease Activity index for PSoriatic Arthritis; DAS28, Disease Activity Score based on 28 joints; ESR, erythrocyte sedimentation rate; HAQ, Health Assessment Questionnaire; mHAQ, modified Health Assessment Questionnaire; RADAI, Rheumatoid Arthritis Disease Activity Index; RAID, Rheumatoid Arthritis Impact of Disease; SF-36, 36-Item Short Form Health Survey

Disease-specific data items for IRDs covered by all registers (RA, PsA, SpA) are presented in Fig. 3. For RA, data is collected quite homogeneously comprising the immunologic markers anti-CCP antibodies and rheumatoid factor, the 28 swollen and tender joint counts, and the composite score DAS28-CRP. No concordance within all four registers was observed for any of the patient-reported outcomes; for example, the Health Assessment Questionnaire (HAQ) was captured either in the original or in the modified version. Regarding PsA and SpA, agreement in data captured by all four registers was found in the reporting of HLA-B27 antigen and the patient-reported disease activity index BASDAI.

### Baseline characteristics and number of patients enrolled in the participating registers of EuNeP

To date, a total of 3504 patients have been enrolled in the 4 European registers contributing 2940 pregnancies. Of those, 2246 pregnancies have been completed (Table 3). The mean age of women ranged from 31 to 33 years with a disease duration of 6 to 10 years. The mean body mass index (BMI) was 24 to 25 kg/m^2^, and 5 to 16% of the patients were obese with a BMI higher than 30 kg/m^2^. Participants had their first visit during pregnancy on average at gestation week 11 (EGR), 13 (Rhekiss and RevNatus), and 19 (RePreg). While 27% of the patients in EGR2 were nulliparous, this applied to 35% in RevNatus, 37% in Rhekiss, and 46% in RePreg. The extent of missing data varied considerably depending on the variable and within the registers. The lowest percentage of missing values for characteristics of Table 3 were reported for age (1 to 13%) and gestational age (0.1 to 13%). More missing values were observed in patient-reported data such as BMI (15 to 61%) and smoking habits (12 to 71%; details on missing data not shown).

**Table 3:** Information on patients/pregnancies at the first visit during pregnancy

	EGR2 (FR)	RePreg (CH)	RevNatus* (NO)	Rhekiss (DE)
Database closed on	17 February 2019	01 February 2019	18 February 2019	03 January 2019
Number of patients	1084	82	724	911
Number of pregnancies	1177	83	861	976
Age, mean (SD)	32.0 (5.0)	32.8 (4.4)	30.9 (4.7)	32.5 (4.3)
BMI, mean (SD)	24.0 (5.1)	25.0 (5.4)	23.9 (7.5)	24.1 (4.9)
BMI > 30, *n* (%)	118 (10.0)	4 (9.3)	133 (16.3)	45 (4.6)
Smoking, current, *n* (%)	97 (9.4)	2 (8.3)^#^	20 (2.4)^#^	23 (4.7)
Smoking, past, *n* (%)	186 (18.0)	n.a.^$^	n.a.^$^	189 (38.7)
Smoking, never, *n* (%)	750 (72.6)	22 (91.7)^#^	818 (97.6)^#^	276 (56.6)
Gestational week at inclusion, mean (SD)	11.0 (6.0)	18.9 (9.5)	13.0 (6.6)	12.9 (5.1)
Nulliparous, *n* (%)	317 (26.9)	35 (45.5)	297 (34.5)	357 (36.6)
Disease duration, mean (SD)	6.1 (6.1)	10.0 (7.0)	9.5 (7.5)	8 (6.9)

Percentages are given for those pregnancies with available data. *Abbreviations*: *BMI* body mass index, *n.a.* not available, *SD* standard deviation

*Only patients enrolled in the IT-based system since March 2016 are considered for the analyses

^#^During the last 3 months

^$^Smoking variable is categorized as smoking/not smoking

## Discussion

Within our study, we collected information on the design and content of data among four European multicentre registers collaborating in EuNeP observing women with several underlying IRDs before, during, and after a pregnancy. This work gives an overview of the unique features of the individual registers and assesses their comparability with each other.

More than 3500 patients have been enrolled in the registers so far, capturing approximately 2200 completed pregnancies although most of these registers have only been established in recent years. This illustrates the magnitude and importance of reproductive issues associated with rheumatic diseases in daily rheumatologic care for health care providers and patients.

A variety of research questions need to be answered by pregnancy registers: What is the impact of pregnancy on the course of IRD? And vice versa: Does the underlying chronic disease affect the course of pregnancy and its outcome? What effect do anti-rheumatic drugs have on the foetus and infant? So far, the ‘oldest’ of the four registers—the Norwegian RevNatus—has answered some of the research questions for PsA, JIA, and SLE [7–11]. However, even if a register has included a considerable number of patients, as soon as the patients are stratified by certain diseases, the decreasing number of patients limits possible investigations. Furthermore, to investigate rare events, e.g. malformations, or the course of pregnancy in rare diseases such as autoinflammatory syndromes, one register might not yield enough information to gain robust results. Therefore, collaboration among registers is desirable.

A prerequisite for collaborative analyses is information on the nature and extent of the data collected in the individual participating registers. However, as a requirement, data items and the way they are assessed need to be congruent to enable comparisons of drug exposures and outcomes across registers.

In principle, the study design of the investigated registers is similar: all the registers collect data prospectively and enrol women with different IRDs. Data is collected throughout the period of child wish, during pregnancy, and after pregnancy, but the lengths of the periods under observation vary. While the period of child wish is recorded for a maximum of 6 months in one register, it may last up to 96 months in another one. The postpartum follow-up periods for live born infants range from 12 to 48 months. All registers include women with RA, PsA, and SpA. Additionally, three registers collect data on other diseases, especially SLE and other connective tissue diseases. However, the distribution of diagnoses among enrolled patients varies. More than half of the patients participating in RevNatus and Rhekiss have a diagnosis of RA, PsA, or SpA compared to only 17% of patients in the French EGR2 register. The majority of EGR2 patients (49%) have a diagnosis of SLE or other connective tissue diseases compared to 20% (RevNatus) and 34% (Rhekiss). This is likely due to differences in the participating study centres across countries, and different health systems and care structures.

With respect to data collection, concordances were found especially regarding maternal demographic information, comorbidities, parity, selected patient-reported outcomes, and neonatal outcomes. Notably, all registers report pregnancy losses, severe pregnancy complications, and major birth defects. In addition, all registers focus on information regarding disease activity and severity, as well as on treatment details such as exposure to DMARDs, glucocorticoid use, and their dosage. We found that on the one hand, there is high consistency regarding the areas on which information is collected, but on the other hand, we revealed considerable differences in how this information is collected, e.g. who is reporting (physician or patient), which instruments or which categories of variables are used. In addition, the time points and frequency of data collection varied. This variability hinders not only the comparability of reported results but also the implementation of collaborative approaches. Andreoli et al. recently reviewed indices used in pregnancy studies and showed a broad range in tools for active disease, flares, remission, and other disease activity measures [12]. This was also observed in our study, but we found that where recommendations for the assessment of disease activity exist, disease-specific data items showed a greater concordance between registers. This is the case for RA in general [13] as well as for pregnant women with RA in prospective studies [14] and was mirrored by a greater agreement of variables collected for patients with RA than those for patients with PsA and SpA.

Harmonization can be achieved by various means, including standardization of datasets, outcome measures, or analysis methods. In rheumatology, several initiatives strengthened the importance of harmonized data collection, and core datasets for observational studies in RA have been developed [15–18]. The need for harmonization of data collection also applies to pregnancy data in rheumatology. To facilitate future data collection and joint approaches, which are important to gain more power of the analyses, a core dataset with a minimum of key items to be collected is important. This would harmonize data collection across the four registers presented in this overview and provide a basis for parties wishing to establish their own national pregnancy registers. To include as many registers as possible, those data should be easy to collect, not being specific for one health system. However, the challenge for collaborative analyses goes beyond data harmonization. Our summary reveals that addressing research questions by collaborative analyses of pregnancy registers will pose multifaceted challenges. Not only the problem of using different assessment tools and follow-up intervals for data collection needs to be solved, but also the issue of missing data of important variables [19]. In addition, very ‘basic’ issues need to be addressed. For instance, the time of baseline must be clearly defined—at least, it should be clearly reported. Depending on the research question, patients enrolled during pregnancy could be eligible or not. For the investigation of early miscarriages, patients should have been enrolled when they wish to become pregnant. Of note, characteristics strongly influenced by pregnancy, such as weight or fatigue, cannot be compared with each other when reported at different time points. It might therefore be necessary to select patient groups, e.g. those enrolled during the first trimester of pregnancy, and define ‘baseline’ in detail, e.g. as the time point of the first visit during pregnancy.

A limitation of this study is the concentration on the four registers that participate in the EuNeP collaboration. In the meantime, more pregnancy registers were established in other European countries, which are now in their first year of data collection. Future cooperation with all registers is very much desired and planned. Thus far, we have not investigated the clinical differences of patients within the registers—a parameter that will influence the search for appropriate analysis methods.

It was our concern to report on the status quo of European registers in the field of pregnancy and IRD, which participate in EuNeP, and to communicate their existence to the international rheumatologic community. These results are a starting point for understanding how to improve data collection in pregnancy registers and pave the way towards further steps such as establishing a core dataset and a first collaborative analysis that includes finding the most suitable approaches for data analysis.

## Conclusions

Although the registers in this collaboration have similar designs, we found numerous differences in the data items collected. In particular, the level of details of documented data and the information on disease-specific characteristics showed heterogeneity. A harmonization of the data collected across pregnancy registers would enable and facilitate comparative analyses. To be able to do this, key items of data reported to registers should be defined. The definitions could also guide and help the establishment of new pregnancy registers.

## Supplementary information

**Additional file 1: Table S1.** Details about data collection with respect to the reporting person, the frequency of collection, and where appropriate instruments and/ or categories used.

## Data Availability

Data of the register survey are available from the corresponding author upon reasonable request. The data presented in Tables 2 and 3 and Fig. 1 were provided by the individual registers.

## Abbreviations

AE: Adverse event
BMI: Body mass index
DMARD: Disease modifying anti-rheumatic drug
EULAR: European League Against Rheumatism
EuNeP: European Network of Pregnancy Registers in Rheumatology
IRD: Inflammatory rheumatic disease
JIA: Juvenile idiopathic arthritis
NIH: National Institutes of Health
NSAID: Non-steroidal anti-inflammatory drugs
PsA: Psoriatic arthritis
RA: Rheumatoid arthritis
SCQM: Swiss Clinical Quality Management in Rheumatic Diseases
SLE: Systemic lupus erythematosus
SpA: Spondyloarthritis
