# Supplementary material for: European Network of Pregnancy Registers in Rheumatology (EuNeP)—an overview of procedures and data collection
Source: Arthritis Res Ther. 2019 Nov 14;21:241. doi: 10.1186/s13075-019-2019-3 (PMC6854642; doi:10.1186/s13075-019-2019-3)
Supplement: Supplementary file 1 — Additional file 1: Table S1. Details about data collection with respect to the reporting person, the frequency of collection, and where appropriate instruments and/ or categories used. [file 13075_2019_2019_MOESM1_ESM.docx]

Supplement - Table 1: Details about data collection with respect to the reporting person, the frequency of collection, and where appropriate instruments and/ or categories used.

| **Data items** | **No. of regi-sters** | **Reported by** | | **Frequency** | | | **Instruments** | **Categories etc.** |
| --- | --- | --- | --- | --- | --- | --- | --- | --- |
|  |  | **Physi-cian** | **Patient** | **Once** | **All visits** | **Selected visits** |  |  |
| Maternal information & demographics |  |  |  |  |  |  |  |  |
| Age | 4 | 4 | 1 | 4x BL |  |  | 4x Date of birth  1x Year of birth |  |
| Inflammatory rheumatic disease |  |  |  |  |  |  |  |  |
| Physician confirmed diagnosis | 4 | 4 |  | 4x BL |  |  | Confirmation by physician |  |
| Confirmation of classification criteria | 4 | 4 |  | 4x BL |  |  | Depending on IRD |  |
| Disease duration | 4 | 4 |  | 4x BL |  |  | 3x Year  1x Month/year |  |
| Symptom duration | 1 | 1 |  | 1x BL |  |  | 1x Year |  |
| Body composition |  |  |  |  |  |  |  |  |
| Body weight | 4 | 3 | 2 |  | 4x ALL |  |  |  |
| Body height | 4 | 3 | 2 | 3x BL | 1x ALL |  |  |  |
| Body mass index | 4^1^ | 3 | 2 |  | 4x ALL |  |  |  |
| Socio-demographic information |  |  |  |  |  |  |  |  |
| Are of residence | 1 | 1 |  | 1x BL |  |  | Postal code |  |
| Family status | 3 | 1 | 2 | 2x BL | 1x ALL |  | 3x Categories | Categories: Living with partner // Living with partner/ Living with another person than a partner/ Living alone // single/ married/ divorced/ separated/ permanent partnership/ widowed |
| Educational level | 3 |  | 3 | 3x BL |  |  | 3x Categories | Categories: Compulsory/ Vacational/ University // Primary school/ Secondary level including high school/ Lower university degree (until 4 years)/ Higher university degree (>4 years) // Highest educational level: No/ 9 years/ 10 years/ 12 years |
| Professional training | 2 |  | 2 | 2x BL |  |  | 2x Categories | Categories: None/ 'learning by doing'/ traineeship/ university education // Not indicated by 1 register |
| Employment/ Work situation | 4 | 1 | 3 | 3x BL | 1x ALL |  | 1x Employment Y/N + 4x Categories | Categories: Farmer/ craftswoman, shopkeeper, business owner/ liberal profession, manager/ intermediate profession/ employee // Full time/ Part time/ Unemployed // Fulltime/ Part time/ Not working related to (I) disease, (II) other reasons, (III) pregnancy leave, (IV) pregnancy money, (V) student // full time/ part time/ professional training or school/ Sick leave/ Unemployed/ Pensioned |
| Sick leave | 3 |  | 3 | 1x BL | 2x ALL |  | 2x Y/N, Not indicated by 1 register |  |
| Ethnicity | 1 | 1 |  | 1x BL |  |  | 1x Categories | Categories: European, Asian, African, Other, Unknown |
| Smoking & alcohol |  |  |  |  |  |  |  |  |
| Smoking habits | 4 | 1 | 3 | 1x BL | 3x ALL |  | 4x Categories  1x No. cigarettes/d  1x Years of smoking  1x Month/Year of quitting | Categories: Never/ current/ past // Smoking/ not smoking // Smoking/ not smoking // Never/ Occasionally/ Currently < 5/day/ Currently > 5/day/ Formerly |
| Alcohol consumption | 3 | 1 | 2 | 1x BL | 1x ALL | 1 | 3x Categories  1x Kind and amount of alcohol, and frequency | Categories: Never/ Current / Past // Alcohol consumption/ no alcohol consumption // Never/ Occasionally/ Regularly |
| Maternal medical treatment |  |  |  |  |  |  |  |  |
| (cs/b/ts)DMARD history |  |  |  |  |  |  |  |  |
| DMARD use | 4 | 4 |  | 3x BL | 1x ALL |  |  |  |
| Drug name | 4 | 4 |  | 3x BL | 1x ALL |  |  |  |
| Dosage | 3 | 3 |  | 1x BL | 1x ALL |  |  |  |
| Start & stop dates | 3 | 3 |  | 1x BL | 1x ALL |  |  |  |
| Current (cs/b/ts)DMARD use |  |  |  |  |  |  |  |  |
| DMARD use | 4 | 4 |  |  | 4x ALL |  |  |  |
| Drug name | 4 | 4 |  |  | 4x ALL |  |  |  |
| Dosage | 4 | 4 |  |  | 4x ALL |  |  |  |
| Application interval | 4 | 4 |  |  | 4x ALL |  |  |  |
| Start & stop dates | 4 | 4 |  |  | 4x ALL |  |  |  |
| Oral glucocorticoids |  |  |  |  |  |  |  |  |
| Use of oral glucocorticoids | 4 | 4 |  | 3 | 1x ALL |  |  |  |
| Drug name | 2 | 2 |  | 2 |  |  |  |  |
| Dosage | 4 | 4 |  | 3 | 1x ALL |  |  |  |
| Application interval | 3 | 3 |  | 2 | 1x ALL |  |  |  |
| Start & stop dates | 2 | 2 |  | 2 |  |  |  |  |
| Intraarticular glucocorticoids |  |  |  |  |  |  |  |  |
| Use of intraarticular glucocorticoids | 4 | 4 |  |  | 3x ALL | 1 |  |  |
| Dosage | 2 | 2 |  |  | 2x ALL |  |  |  |
| Application date | 2 | 2 |  |  | 2x ALL |  |  |  |
| NSAIDs, Analgesics, Opioids |  |  |  |  |  |  |  |  |
| Use of NSAIDs, Analgesics, Opioids | 4 | 4 |  |  | 4x ALL |  |  |  |
| Drug name | 3 | 3 |  |  | 3x ALL |  |  |  |
| Dosage | 2 | 2 |  |  | 2x ALL |  |  |  |
| Application interval | 1 | 1 |  |  | 2x ALL |  |  |  |
| Start & stop dates | 4 | 4 |  |  | 4x ALL |  |  |  |
| Comedication |  |  |  |  |  |  |  |  |
| Use of comedication | 4 | 4 | 1 |  | 4x ALL |  |  |  |
| Kind of drug | 4 | 3 | 1 |  | 4x ALL |  | 4x Predefined list  1x Free text | Predefined lists encompass drug groups and differ from each other. |
| Maternal health status |  |  |  |  |  |  |  |  |
| Comorbidities & adverse events |  |  |  |  |  |  |  |  |
| Comorbidities | 4 | 4 |  |  | 3x ALL | 1 | 4x Predefined list  1x Free text | Predefined lists differ from each other. All registers capture information on pre-gestational diabetes, Hypertension/CVD, psychiatric disorders, thyroid disorders |
| Adverse events | 4 | 4 |  |  | 4x ALL |  | 3x Predefined list  2x Free text  2x start date  2x seriousness of event | Predefined lists differ from each other |
| Hospital admission | 3 | 2 | 1 |  | 3x ALL |  | 1xY/N  1x categories  1x by linkage  1x Indication of length of stay | Categories: Hospitalization/ prolonged hospitalization |
| IRD outcomes |  |  |  |  |  |  |  |  |
| Global disease activity | 4^4^ | 3 | 4 |  | 4x ALL |  | 3x NRS (0-10)  1x NRS (0-3)^6^  1x VAS |  |
| General health status | 3 | 0 | 3 |  | 3x ALL |  | 2x NRS (0-10)  1x SF-12 |  |
| Pain | 4 | 1 | 4 |  | 4x ALL |  | 2x NRS  2x VAS |  |
| Disease severity | 2 | 1 | 1 | 1x BL | 1x ALL |  | 1x Likert scale  1x VAS |  |
| Fatigue | 3 | 0 | 3 |  | 3x ALL |  | 2x NRS  1x VAS |  |
| Lab: Inflammation markers |  |  |  |  |  |  |  |  |
| CRP | 4 | 4 | - |  | 4x ALL |  |  |  |
| ESR | 3 | 3 | - |  | 3x ALL |  |  |  |
| Lab: Blood count |  |  |  |  |  |  |  |  |
| Haemoglobin | 4 | 4 | - |  | 3x ALL | 1^5^ |  |  |
| Thrombocytes | 3 | 3 | - |  | 2x ALL | 1 |  |  |
| Leucocytes | 2 | 2 | - |  | 2x ALL |  |  |  |
| Lymphocytes | 1 | 1 |  |  | 1x ALL |  |  |  |
| Lab: Kidney and hepatic function |  |  |  |  |  |  |  |  |
| Creatinine | 3 | 3 | - |  | 2x ALL | 1 |  |  |
| ALAT (Alanine aminotransferase) | 2 | 2 | - |  | 2x ALL |  |  |  |
| GFR ( Glomerular filtration rate | 1 | 1 | - |  | 1x ALL |  |  |  |
| ALP (Alkaline phosphatase) | 1 | 1 | - |  | 1x ALL |  |  |  |
| Lab: Other markers and tests |  |  |  |  |  |  |  |  |
| Urinary analysis | 3 | 3 | - |  | 3x ALL |  |  |  |
| Uric acid | 2 | 2 | - |  | 1x ALL | 1 |  |  |
| Immunoglobulin G | 2 | 2 | - |  | 2x ALL |  |  |  |
| Pregnancy |  |  |  |  |  |  |  |  |
| Details about previous pregnancies |  |  |  |  |  |  |  |  |
| No. of previous pregnancies | 4 | 4 |  | 4x BL |  |  |  |  |
| Outcome of previous pregnancies | 4 | 4 |  | 4x BL |  |  |  |  |
| Pregnancy complications | 4 | 4 |  | 3x BL |  |  |  |  |
| Year of birth | 3 | 3 |  | 3x BL |  |  |  |  |
| Details about current pregnancy |  |  |  |  |  |  |  |  |
| Calculated date of birth | 4 | 4 |  | 3x GE | 1x ALL |  | 4x Menstruation based  3x Ultrasound based  1x EDD based |  |
| Use of pre-pregnancy counselling | 4 | 3 | 2 | 1x BL 3x GE |  |  | 3x Y/N  1x Categories | Categories: Physician-CRF: No/ by rheumatologist/ by gynaecological outpatient clinic/ genetic counselling;  Patient-CRF: No/ by gynaecologist/ by obstetrician/ genetic counselling/ counselling for clotting disorder |
| Planning of pregnancy | 4 | 3 | 2 | 1x BL 3x GE |  |  | 2x Y/N  1x Indication of months,  Not indicated by 2 registers |  |
| Use of reproductive medicine | 3 | 3 | 1 | 1x BL 2x GE |  |  | 2x Y/N  1x Categories  2x Indication of method | Categories: Yes, only councelling/Yes, with diagnostic/ Yes, with treatment/ No;  Methods: Insemination/ IVF/ ICSI/ other // Medicinal/ operational/ insemination/ IVF/ ICSI/ unknown |
| Indication of multiple pregnancies | 4 | 4 |  | 2x GE 1x PP |  | 1 | 2x No. of foetuses  1x Categories  Not indicated by 1 register | Categories: No/ 2 foetuses/ 3 foetuses |
| Use of contraceptives prior conception | 2 | 1 | 1 | 2x GE |  |  | 2x Y/N  2x Categories | Categories: Pill/ patch/ implanon/ Depot provera/ Nuvaring/ IUD (copper, progesterone)/ condome // Hormonal/ intrauterine device/ barrier |
| Course of pregnancy |  |  |  |  |  |  |  |  |
| Maternal BP | 4 | 4 |  |  | 4x ALL |  |  |  |
| Fetal malformations | 4 | 4 |  | 1x PP | 3xGE |  | 2x Y/N  3x free text  1x by linkage |  |
| Ultrasound screening | 2 | 2 |  |  | 2x GE |  | 2x categories | Categories: Normal/ abnormal (+ additional information if abnormal ultrasound) // Categories differ depending on WGA and are according to national recommendations |
| Normal intrauterine growth | 2 | 2 |  |  | 2x GE |  | 2x Y/N |  |
| Pregnancy complications |  |  |  |  |  |  |  |  |
| (Pre-)Eclampsia | 4 | 4 |  | 2x PP | 1x GE | 1 | 4x Y/N  1x Indication of symptoms | Symptoms: Hypertension/ Proteinuria/ Oedema/ Cerebral involvement |
| HELLP syndrome | 4 | 4 |  | 2x PP | 1x GE | 1 | 4x Y/N |  |
| Gestational diabetes | 3 | 3 |  |  | 2x GE | 1 | 3x Y/N |  |
| Infections | 3 | 3 |  |  | 2x GE | 1 | 3x Y/N |  |
| Thromboembolic events | 3 | 3 |  |  | 2x GE | 1 | 3x Y/N |  |
| Arterial hypertension | 2 | 2 |  |  | 1x GE | 1 | 2x Y/N |  |
| Pregnancy outcomes |  |  |  |  |  |  |  |  |
| Gestational age at birth | 4 | 4 | 0 | 4x PP |  |  |  |  |
| Elective termination | 4 | 4 | 1 | 4x PP |  |  | 2x categories  Not indicated by 2 registers | Categories: Induced abortion/ medical termination (with the reasons) // Maternal wish (until WGA12)/ Medically indicated/ Criminological |
| Pregnancy loss | 4 | 4 | 1 | 4x PP |  |  | 3x categories  Not indicated by 1 register | Categories: Spontaneous miscarriage/ Ectopic pregnancy/ stillbirth // Spontaneous abortion< WGA 12/ spontaneous abortion WGA 12-21/ Stillbirth week 12 onwards // Spontaneous abortion (WGA<20)/ Intrauterine death (WGA>20)/ Abortion (<500g)/ Stillbirth (>500g) |
| Live birth | 4 | 4 | 0 | 4x PP |  |  |  |  |
| Mode of delivery | 4 | 4 | 1 | 4x PP |  |  | 4x Categories  4x Reasons for CS | Categories: Spontaneous/ elective CS/ emergency CS // spontaneous/ elective CS/ emergency CS // spontaneous/ elective CS/ emergency CS/ operative delivery (vacuum, forceps) // spontaneous/ elective CS/ emergency CS/ operational vaginal |
| Outcomes of the child |  |  |  |  |  |  |  |  |
| Socio-demographic information |  |  |  |  |  |  |  |  |
| Gender | 4 | 4 |  | 4x PP |  |  |  |  |
| Body weight | 4 | 3 | 2 | 2x PP | 2x PP |  |  |  |
| Body height | 3 | 2 | 2 | 1x PP | 2x PP |  |  |  |
| Breastfeeding of the child | 4 | 3 | 2 | 1x PP | 2x PP | 1 | 1x Y/N  1x categories  2x date of cessation  Not indicated by 2 registers | Categories: Currently breastfeeding/ Never breastfeeding/ terminated breastfeeding |
| Health-related information |  |  |  |  |  |  |  |  |
| Apgar | 3 | 2 | 1 | 3x PP |  |  | 2x Apgar 5 and 10  Not indicated by 1 register |  |
| Diseases | 3 | 3 | 1 |  | 3x PP |  | 2x predefined list  2x free text  Not indicated by 1 register |  |
| Neonatal malformations | 3 | 4 | 0 | 3x PP |  |  | 3x free text  1x by linkage |  |
| Vaccination | 3 | 2 | 1 |  | 1x PP | 2 | 2x Predefined list  1x free text | List: MMR vaccine/ BCG vaccine // List according to national vaccination recommendations |
| Hospital admission | 2 | 2 | 1 |  | 2x PP |  | Not indicated by 2 registers |  |
| Medical treatment | 1 | 0 | 1 |  | 1x PP |  | 1x free text |  |

*ALL: data is captured at all visits. BL: Data is captured at the baseline visit/ at inclusion. GE: data is captured once at the first gestational visit (Column: Frequency - once), or at every gestational visit (Column: Frequency - all visits). PP: Data is captured at the first postpartal visit (Column: Frequency - once), or at every postpartal visit (Column: Frequency - all visits).*

*Abbreviations:* *CS, Caesarean section; d, day; EDD, Estimated Date of Delivery (EDD) Pregnancy Calculator; No., number; NRS, numeric rating scale; SF-12, Short form 12 health Questionnaire; VAS, visual analogue scale; WGA, week gestational age.*

*^1^In two registers, BMI is not directly reported but can be calculated.*

*^2^Adverse events are registered as change in comorbidity in one register.*

*^3^One register does not capture the data but can get information through linkage to other registers.*

*^4^Only for selected diseases in one register.*

*^5^Only if indicated.*

*^6^For lupus activity.*
